# Mitochondrial 2,4-dienoyl-CoA Reductase Deficiency in Mice Results in Severe Hypoglycemia with Stress Intolerance and Unimpaired Ketogenesis

**DOI:** 10.1371/journal.pgen.1000543

**Published:** 2009-07-03

**Authors:** Ilkka J. Miinalainen, Werner Schmitz, Anne Huotari, Kaija J. Autio, Raija Soininen, Emiel Ver Loren van Themaat, Myriam Baes, Karl-Heinz Herzig, Ernst Conzelmann, J. Kalervo Hiltunen

**Affiliations:** 1 Department of Biochemistry and Biocenter Oulu, University of Oulu, Oulu, Finland; 2 Theodor-Boveri-Institut für Biowissenschaften (Biozentrum) der Universität Würzburg, Würzburg, Germany; 3 Department of Biotechnology and Molecular Medicine, A.I. Virtanen Institute for Molecular Sciences, Kuopio, Finland; 4 Department of Medical Biochemistry and Biocenter Oulu, University of Oulu, Oulu, Finland; 5 Bioinformatics Laboratory, Department of Clinical Epidemiology, Biostatistics and Bioinformatics, Academic Medical Center, Amsterdam, The Netherlands; 6 Laboratory of Cell Metabolism, Department of Pharmaceutical Sciences, Katholieke Universiteit Leuven, Leuven, Belgium; 7 Department of Internal Medicine, Kuopio and Institute of Biomedicine, Division of Physiology and Biocenter of Oulu, Oulu University Medical School, Oulu, Finland; Burnham Institute for Medical Research, United States of America

## Abstract

The mitochondrial β-oxidation system is one of the central metabolic pathways of energy metabolism in mammals. Enzyme defects in this pathway cause fatty acid oxidation disorders. To elucidate the role of 2,4-dienoyl-CoA reductase (DECR) as an auxiliary enzyme in the mitochondrial β-oxidation of unsaturated fatty acids, we created a DECR–deficient mouse line. In *Decr^−/−^* mice, the mitochondrial β-oxidation of unsaturated fatty acids with double bonds is expected to halt at the level of *trans*-2, *cis/trans*-4-dienoyl-CoA intermediates. In line with this expectation, fasted *Decr^−/−^* mice displayed increased serum acylcarnitines, especially decadienoylcarnitine, a product of the incomplete oxidation of linoleic acid (C_18:2_), urinary excretion of unsaturated dicarboxylic acids, and hepatic steatosis, wherein unsaturated fatty acids accumulate in liver triacylglycerols. Metabolically challenged *Decr^−/−^* mice turned on ketogenesis, but unexpectedly developed hypoglycemia. Induced expression of peroxisomal β-oxidation and microsomal ω-oxidation enzymes reflect the increased lipid load, whereas reduced mRNA levels of PGC-1α and CREB, as well as enzymes in the gluconeogenetic pathway, can contribute to stress-induced hypoglycemia. Furthermore, the thermogenic response was perturbed, as demonstrated by intolerance to acute cold exposure. This study highlights the necessity of DECR and the breakdown of unsaturated fatty acids in the transition of intermediary metabolism from the fed to the fasted state.

## Author Summary

Fatty acids released from triacylglycerols or obtained from the diet serve as a main energy provider to the heart and skeletal muscle, and when carbohydrates are scarce, fatty acids provide energy for the whole organism. Inherited disorders of mitochondrial β-oxidation are among the most common inborn errors of metabolism affecting infants and children. Under normal conditions, patients are usually asymptomatic; but when challenged with metabolic stress, severe phenotypes arise. Here we describe the generation of a mouse model in which the total degradation of unsaturated fatty acids is prevented by disruption of an auxiliary enzyme of β-oxidation. Although degradation of saturated fatty acids proceeds normally, the phenotype presented here is in many ways similar to mouse models of the disrupted classical β-oxidation pathway, but with additional unique features. The null mutant mice are asymptomatic until exposed to fasting, during which they switch on ketogenesis, but simultaneously develop hypoglycemia. A number of human patients suffer from idiopathic hypoglycemia (hypoglycemia of unknown cause). Our mouse model links this disease state to a specific defect in the breakdown of polyunsaturated fatty acids. Furthermore, it shows that degradation of unsaturated fatty acids is essential for balanced fatty acid and energy metabolism, as well as adaptation to metabolic stress.

## Introduction

Fatty acids are amphipathic molecules that have indispensable roles in many cellular functions. In addition to energy storage in the form of triacylglycerols, fatty acids are involved in the synthesis of membrane lipids and in signal transduction and endocrine processes. When carbohydrates are depleted as an energy source during fasting and starvation, triacylglycerol stores are mobilized and acetyl-CoAs produced by hepatic β**-**oxidation of fatty acids are condensed to ketone bodies to ensure an alternative fuel source for extrahepatic tissues, such as brain, skeletal muscle, and cardiac muscle.

Inherited disorders of mitochondrial β-oxidation are among the most common inborn errors of metabolism affecting infants and children. Although clinical phenotypes vary, the inability to completely utilize fatty acids during periods of increased energy requirement is common to all ß-oxidation disorders. Under normal conditions, patients are usually asymptomatic, but when challenged with short-term fasting during infectious illness, severe and even fatal phenotypes arise. Disease states can manifest as one or more of the following characteristics: liver dysfunction, hypoketotic hypoglycemia, organic aciduria, skeletal myopathy, and elevated fatty acid concentrations in the serum and tissues [1].

**Figure 1:**
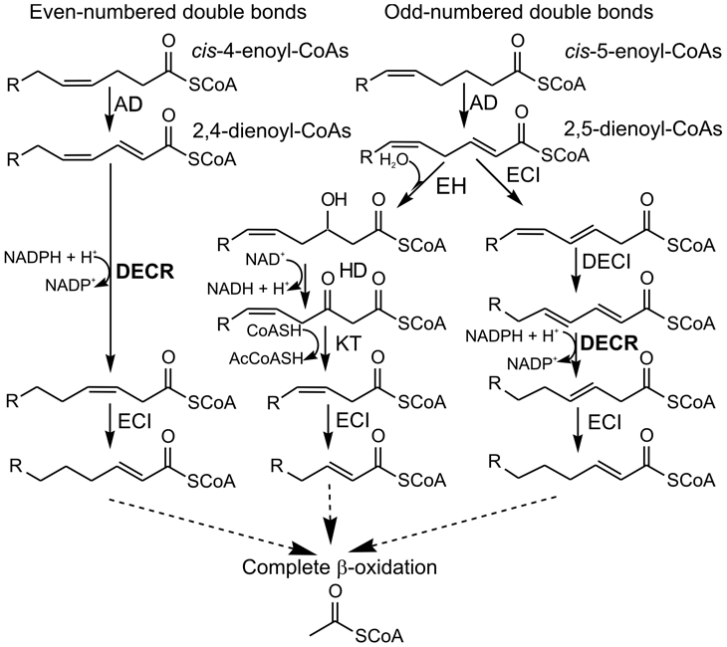
β-oxidation of fatty acids with double bonds at even- or odd-numbered positions in mitochondria. Degradation of fatty acids with even-numbered double bonds results in 2,4-dienoyl-CoA esters, which are oxidized as shown on the left. 2,5-dienoyl-CoA esters arising from odd-numbered double bonds can be oxidized either via an isomerase-dependent pathway (middle) or via a reductase-dependent pathway (right). AD, acyl-CoA dehydrogenase (EC 1.3.3.6, EC 1.3.99.3, EC 1.3.99.13 or EC 1.3.99.-); EH, enoyl-CoA hydratase (EC 4.1.2.17 or EC 4.2.1.74); HD, 3-hydroxyacyl-CoA dehydrogenase (EC 1.1.1.35 or EC 1.1.1.211); KT, 3-ketoacyl thiolase (EC 2.3.1.16) ; ECI, Δ^3^,Δ^2^-enoyl isomerase (EC 5.3.3.8); DECI, Δ^3,5^,Δ^2,4^-dienoyl-CoA isomerase (no EC number available); DECR (shown in bold), 2,4-dienoyl-CoA reductase (EC 1.3.1.34).

The presence of *cis* double bonds in naturally occurring (poly−) unsaturated fatty acids poses problems for ß-oxidation, that require a few auxiliary enzymes (for review, see [2]). During degradation, double bonds in odd-numbered positions (e.g., oleic acid) lead to Δ^3^-enoyl-CoAs, which must be isomerized by an enoyl-CoA isomerase (ECI) (Figure 1, center pathway). Double bonds in even-numbered positions give rise to conjugated Δ^2^,Δ^4^-dienoyl-CoAs, which cannot be hydrated by the enoyl-CoA hydratases for thermodynamic reasons [3]. In eukaryotes, they are reduced by an NADPH-dependent 2,4-dienoyl-CoA reductase (DECR) to 3-enoyl-CoA, which is then isomerized by ECI to *trans*-2-enoyl-CoA, suitable for further oxidation (Figure 1, left pathway). DECR may also play a role in the degradation of fatty acids containing odd-numbered double bonds because the intermediate 2,5-dienoyl-CoA may be isomerized by ECI to 3,5-dienoyl-CoA and then converted to 2,4-dienoyl-CoA by a specific Δ^3,5^,Δ^2,4^-dienoyl-CoA isomerase (Figure 1, right pathway) [4],[5]. In mammals, both mitochondria and peroxisomes contain the full set of these auxiliary enzymes for the breakdown of unsaturated fatty acids [2].

Mammalian mitochondrial isoforms of DECR have been characterized at the nucleotide [6]–[8] and protein level [9]–[13], and the structure of the human 120 kD isoform has been recently solved [14]. Another mitochondrial isoform with a molecular mass of 60 kD has been partially purified, but it has not been characterized at the molecular level.

Although the impact of (poly)unsaturated fatty acids on human well-being has been broadly discussed in both the media and the scientific literature, our understanding regarding the β-oxidation of unsaturated fatty enoyl-CoA esters in a physiological context is limited. Most reported cases of mitochondrial β-oxidation disorders have been related to failures in the oxidation of saturated fatty acids. However, the importance of the complete oxidation of (poly)unsaturated fatty acids for human health has been shown by the case of a patient with a deficiency in mitochondrial DECR activity who died at the age of four months [15].

Excluding Eci null mutant mice [16], the available mouse models address only the breakdown of saturated fatty acids. To study the role of mitochondrial DECR in mammalian metabolism, we generated a mouse model in which *Decr* was disrupted by homologous recombination. Disruption of *Decr* leads to intolerance to fasting, as indicated by hypoglycemia, hepatic microvesicular steatosis, and an altered fatty acid pattern in the liver and serum. Contrary to many other animal models of fatty acid oxidation disorders in which hypoglycemia is associated with hypoketonemia, the absence of DECR activity did not alter the ketogenic response to fasting. A compromised response to stress was also manifested by the inability to maintain a normal body temperature during cold exposure.

## Results

### Generation of *Decr* ^−/−^ Mice

**Figure 2:**
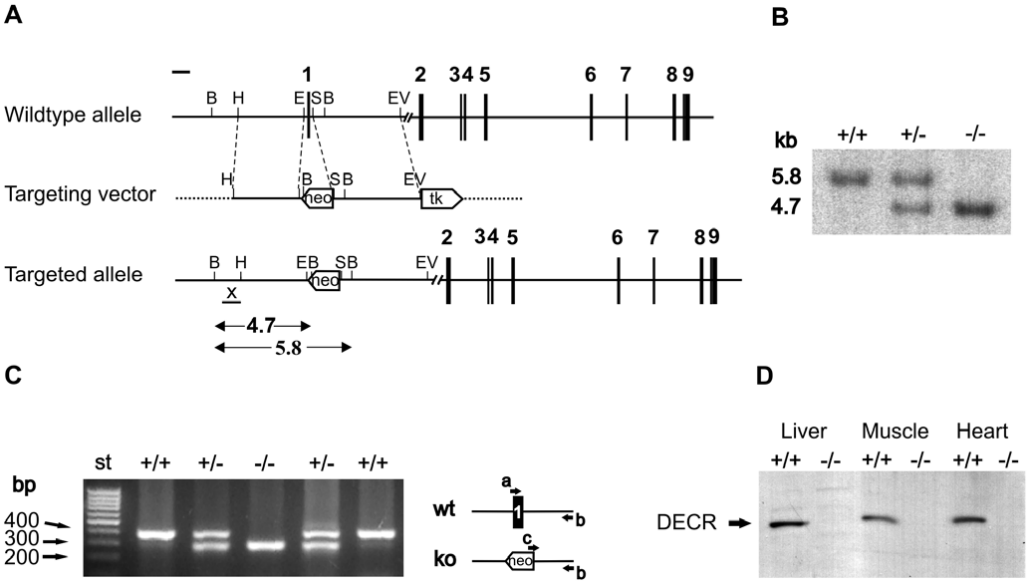
Targeting of the *Decr* locus and verification of gene inactivation. (A) Schematic drawing of the targeting strategy showing the wild type allele, targeting vector and targeted allele after homologous recombination. The targeted allele depicts the strategy used to delete exon 1, in which the endogenous sequence is replaced by a neomycin-positive selection cassette (neo). A thymidine kinase cassette (tk) was used for negative selection. Exons are denoted as numbered solid bars (1–9). Recognition sites for restriction endonucleases are marked as B = *Bam*HI, H = *Hind*III, E = *Eco*RI, S = *Smal*I, EV = *Eco*RV. The genomic fragment used as an external probe for Southern analysis is marked with an “X”, and the expected fragments for the wild type and targeted allele after *Bam*HI digestion are indicated with arrows. (B) Southern blot analysis of mouse liver DNA. Genomic DNA was digested with *Bam*HI and detected using probe X to yield the expected fragments of 5.8 kb for the wild type allele in *Decr^+/+^* mice (+/+), 4.7 kb for the targeted allele in *Decr^−/−^* mice (−/−), and both fragments in heterozygous *Decr^+/−^* mice (+/−). (C) Mouse genotypes were determined from tail samples using PCR with primers denoted with arrows a, b, and c in the schematic drawing. The amplified fragment for wild type *Decr^+/+^* mice was 382 bp, 280 bp for homozygous *Decr^−/−^* mice, and both fragments were amplified from heterozygous *Decr^+/−^* mice. (D) Western blot analysis of mitochondrial homogenates from liver, muscle and heart using an antibody against rat DECR showing the presence or absence of the 33-kDa band corresponding to DECR in *Decr^+/+^* and *Decr^−/−^* mice, respectively. Twenty micrograms of protein was loaded in each lane.

A replacement vector was designed as described under Materials and Methods to replace a 0.5-kb region in the *Decr* locus by homologous recombination. This region containing the first exon of *Decr* was replaced with a *neo* selection cassette in targeted RW4 cells (Figure 2A). Correct targeting was verified by Southern blotting. A 1-kb probe hybridizing to the promoter region of the *Decr* gene, which is not present in the replacement vector, labeled a 5.8-kb fragment in the wild type allele formed after *Bam*HI digestion. Hybridization of the probe to a 4.7-kb fragment of the digested allele from *Decr* ^−/−^ mice confirmed the correct insertion of the *neo* cassette (Figure 2B). Chimeric mice were produced by microinjecting correctly targeted RW4 cells into C57BL/6 blastocysts. Chimeric mice were backcrossed onto C57BL/6 mice to produce *Decr* ^+/−^ and finally *Decr* ^−/−^ mice. The different mouse genotypes were distinguished by PCR using genomic DNA (Figure 2C).

Immunoblotting of mitochondrial extracts from liver, muscle and heart with an antibody against human DECR revealed a detectable signal from wild type mice, whereas no signal could be detected for homozygous null mutant mice (Figure 2D). The reductase activity (n = 3) measured in liver (muscle) mitochondrial extract was 2.2±0.6 µmol/min per mg of protein (2.6±0.3 µmol/min per mg protein) and 0.5±0.1 µmol/min per mg of protein (trace) for wild-type and Decr^−/−^ mice, respectively. It is likely that the observed “residual activity” represents the activity of recently characterized mitochondrial 2-enoyl thioester reductase (EC 1.3.1.38) [17], which functions in mitochondrial fatty acid synthesis and can also reduce 2,4-hexadienoyl-CoA *in vitro* [18].

### Clinical Phenotype

Under standard laboratory conditions, *Decr* ^−/−^ mice were indistinguishable from wild type mice. Crossbreeding of *Decr* ^+/−^ mice produced progeny in approximately Mendelian ratios, with no gender bias (Table 1). Both male and female mutant mice were viable and fertile. They exhibited weight gain and a life-span similar to that of wild type mice. Analysis of organ weights and histological analysis of major organs, including liver, muscle, heart, kidney, lungs, spleen and intestine, showed no differences between wild type and mutant mice.

**Table 1:** Genotype analysis of progeny resulting from crossbreeding *Decr* ^+/−^ mice.

Mouse group	Mice with indicated genotype (% of total)			Mice with indicated gender (% of total)
	+/+	+/−	−/−	
Female	15	32	19	66 (52.0)
Male	14	31	16	61(48.0)
				
Total	29 (23)	63 (49)	35 (28)	127

### Fasting Intolerance

A common feature of individuals affected with inborn errors of mitochondrial fatty acid oxidation is that they are asymptomatic under normal conditions. The same phenomenon is observed in several animal models of fatty acid oxidation disorders. Clinical symptoms arise only after metabolic stress, such as prolonged physical exercise or fasting, which is often associated with infectious illness. In order to study the effect of metabolic stress on *Decr* ^−/−^ mice, the mice were subjected to fasting for 24 or 48 h.

### Altered Lipid Homeostatic Response

**Figure 3:**
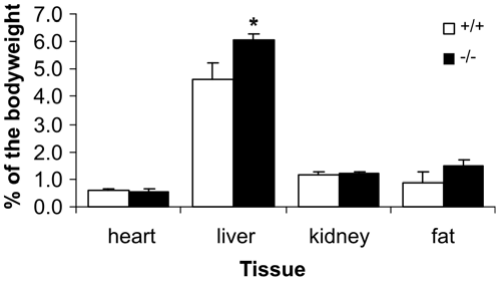
Effects of fasting on organ weights in wild-type and *Decr* ^−/−^ mice. Age-matched male mice were fasted for 24 h, after which they were sacrificed. The wet weight of selected organs (heart, liver, kidney and fat) was determined and compared between wild type and *Decr^−/−^* mice. Weights were calculated as a percentage of body weight and are expressed as means±SE of 4–6 mice of each genotype per group. Asterisks (*) denote significant differences (p<0.01) between wild type and *Decr^−/−^* mice.

**Figure 4:**
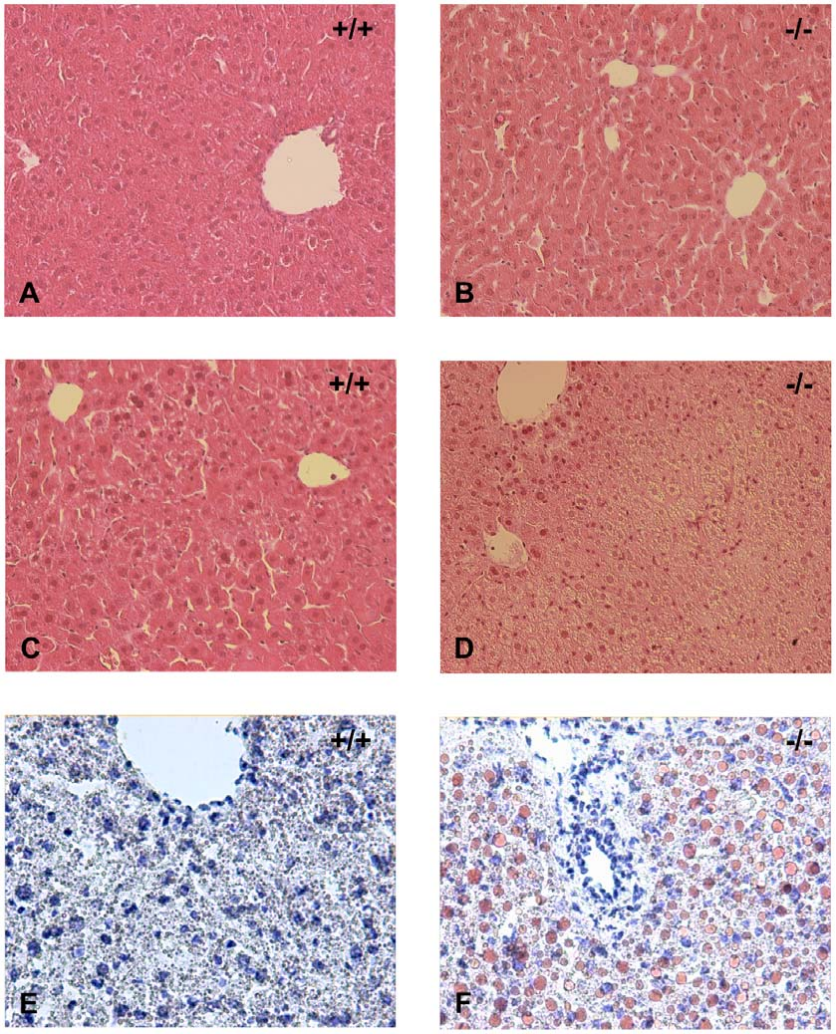
Histological assessment of liver morphology in fasted and non-fasted wild-type and *Decr* ^−/−^ mice. Light microscopic images of representative paraffin-embedded liver sections from wild type (+/+) and *Decr^−/−^* mice (−/−) stained with hematoxylin and eosin (A–D) and cryosections stained with Oil red O (E,F). Liver morphology of non-fasted animals showed no differences between wild type and *Decr^−/−^* mice (A,B). Fasting for 24 h revealed no apparent changes in the liver morphology of wild type mice, but induced microvesicular steatosis in *Decr^−/−^* mice, as observed by the appearance of foamy hepatocytes with centralized nuclei (C,D). Oil red O staining of neutral fat in representative liver cryosections revealed small, lightly stained vacuoles in the wild type sample (E), whereas a large number of intensively stained vacuoles of varying size can be seen in *Decr^−/−^* mice (F), indicating the accumulation of fat. Magnification×20.

During and after fasting, the *Decr* ^−/−^ mice showed a tendency to be more passive and unresponsive compared with wild type mice. Mice were sacrificed and blood and selected organs were collected for further characterization. No differences were observed in the levels of serum alanine aminotransferase, alkaline phosphatase or glutamyl transferase between wild type and *Decr^−/−^* mice, indicating intact liver cells. Concentrations of different amino acids in the sera were also comparable (Table S1). The livers of the *Decr^−/−^* mice were markedly pale, and liver weights, when determined as a percentage of body weight, were significantly (p<0.01) greater than that of wild type mice (Figure 3). Hematoxylin and eosin-stained histological liver sections (Figure 4A and 4B) obtained during the fed state showed no differences. Fasted *Decr^−/−^* mice showed normal lobular architecture when compared with wild type mice, except for the presence of hepatocytes with a foamy appearance and centralized nuclei, which are characteristics of microvesicular steatosis (Figure 4C and 4D). Hepatocytes with extensive microvesicular vacuolation were mainly present in periportal and midzonal regions, whereas the majority of hepatocytes in centrilobular regions appeared normal. When Oil red O staining was performed to stain neutral lipids, the sections showed massive and homogeneously distributed micro- and macrovesicular lipid droplet formation, whereas only minor microvesicular lipid droplet accumulation was present in age-matched wild type control sections (Figure 4E and 4F). These data suggested that fasting results in the accumulation of lipids in the livers of *Decr^−/−^* mice.

**Figure 5:**
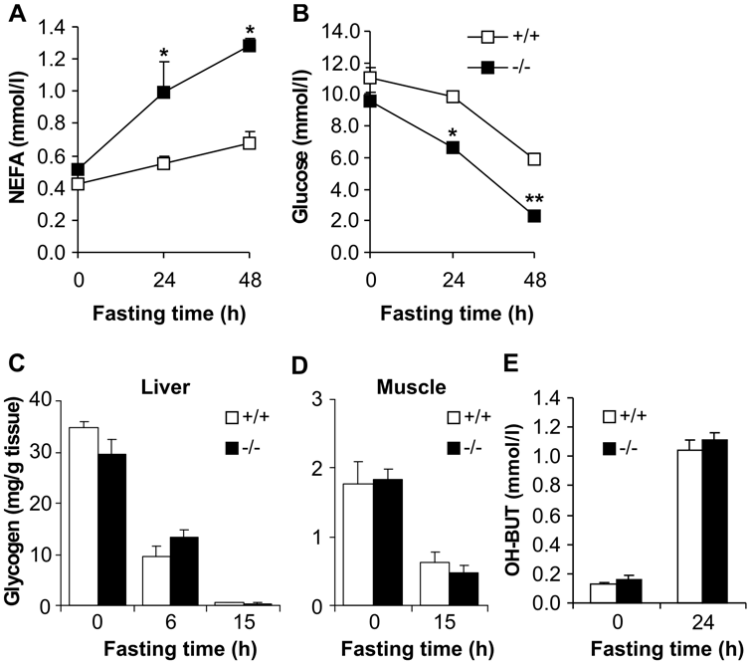
Effect of fasting on serum NEFA, glucose, and OH–BUT levels and liver and muscle glycogen content in wild-type and *Decr^−/−^* mice. Age-matched male wild type (open boxes/bars) and *Decr^−/−^* mice (solid boxes/bars) were fasted for 0, 24, and 48 h, after which the serum levels of non-esterified fatty acids (A) and glucose (B) were determined. Glycogen content of liver (C) and muscle (D) tissue from wild type (open bars) and *Decr^−/−^* mice (solid bars) in the fed state and after mice were fasted for 6 h and/or 15 h was analyzed using the phenol-sulfuric acid method. Serum β-hydroxybutyric acid levels were measured in the fed state and after 24 h of fasting (E). At each time point, the results are expressed as means±SE of 5–6 mice of each genotype per group. Significant differences in glucose and NEFA concentrations between wild type and *Decr^−/−^* mice are indicated by asterisks (* p<0.05, ** p<0.01).

The amounts of circulating non-esterified fatty acids (NEFA) were analyzed in the sera of *Decr^−/−^* and wild type mice. Under normal nutritional conditions, mean serum NEFA levels were comparable between wild type and *Decr^−/−^* mice (0.43±0.11 mmol/l, 0.52±0.03 mmol/l, respectively) but after fasting, the *Decr^−/−^* mice demonstrated increased serum NEFA levels, reaching 1.28±0.12 mmol/l after 48 h compared with the wild type levels of 0.68±0.16 mmol/l (p<0.001) (Figure 5A).

### Altered Glucose Homeostatic Response

A common symptom associated with inherited defects of mitochondrial fatty acid oxidation is the development of hypoglycemia in response to fasting, a phenomenon also observed in several animal models of disrupted mitochondrial fatty acid oxidation [19]–[21]. This effect is considered to be caused by glycogen depletion in combination with an impaired gluconeogenic response. In order to analyze whether the defect in mitochondrial oxidation of (poly)unsaturated fatty acids generates a similar hypoglycemic condition, serum glucose levels were determined for wild type and *Decr^−/−^* mice after 24 h and 48 h of fasting (Figure 5B). In the fed state, glucose levels were comparable (11.0±1.4 mmol/l for wild type and 9.5±0.6 mmol/l for *Decr^−/−^* mice). Twenty-four-hour fasting had no effect on the serum glucose levels of wild type mice (10.9±0.3 mmol/l), whereas a significant decrease was observed in the levels in *Decr^−/−^* mice (6.6±0.2 mmol/l, p<0.01). After mice were subjected to prolonged fasting (48 h), the glucose levels in *Decr^−/−^* mice were further decreased to 2.3±0.3 mmol/l, whereas the decrease in wild type mice resulted in a glucose concentration of 5.9±0.9 mmol/l. These data revealed that *Decr^−/−^* mice have an accelerated hypoglycemic response to fasting.

In order to determine whether the hypoglycemic state of the *Decr^−/−^* mice after fasting is in part due to more rapid depletion of glycogen stores, the liver and muscle glycogen concentration was measured before and after 6 h, 15 h, and 24 h of fasting (Figure 5C and 5D). In the fed state, liver and muscle glycogen content was similar between wild type and *Decr^−/−^* mice. As expected, fasting resulted in a gradual depletion of glycogen stores, and no significant differences in glycogen content between wild type and *Decr^−/−^* mice were found at any observation time points.

Hypoketonemia is often associated with fasting-induced hypoglycemia and defective mitochondrial fatty acid oxidation. The hypoketotic state is caused by an inability of mitochondria to offer enough acetyl-CoA moieties (products of β-oxidation) for ketone body production during fasting. Hypoketotic hypoglycemia is a condition that is used in the diagnosis of human genetic defects to establish a link between symptoms and a fatty acid oxidation defect. To study the ketogenic response to fasting, serum β-hydroxybutyrate levels were measured in *Decr^−/−^* and wild type mice under the fed state and after 24 h of fasting (Figure 5E). In the fed state, the formation of ketone bodies was very low, as indicated by values of 0.13±0.01 mmol/l and 0.16±0.06 mmol/l for wild type and *Decr^−/−^* mice, respectively. Fasting greatly increased serum β-hydroxybutyrate values in the wild type and *Decr^−/−^* mice and comparable values of 1.04±0.14 mmol/l for wild type and 1.11±0.10 for *Decr^−/−^* mice indicated that the reduced capacity for the mitochondrial oxidation of (poly)unsaturated fatty acids in *Decr^−/−^* mice did not prevent a normal ketogenic response.

### Altered Fatty Acid Pattern of Total Lipids

**Figure 6:**
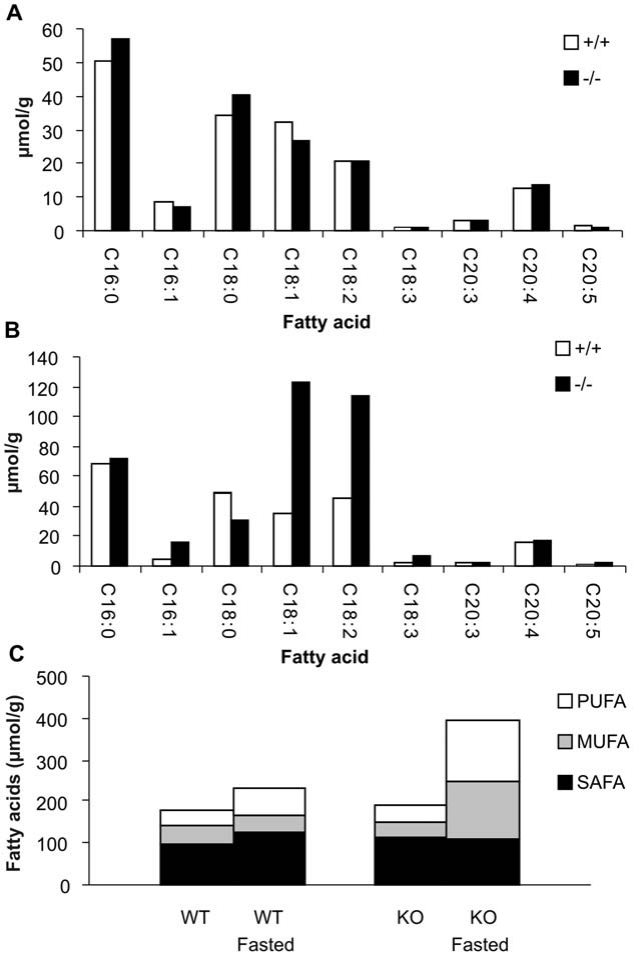
Fatty acid pattern of total liver lipids in fasted and non-fasted wild-type and *Decr^−/−^* mice. Total fatty acids were isolated from pooled liver homogenate samples of 5–6 mice per genotype and analyzed using positive ion mass spectrometry. (A) Fatty acid profile of total liver fatty acids under normal fed state conditions showing the concentrations of different fatty acids for wild type (open bars) and *Decr^−/−^* (solid bars) mice. (B) Fatty acid profile of liver total fatty acids after fasting for 24 h showing increased concentrations of unsaturated fatty acids in *Decr^−/−^* mice when compared with wild type mice. (C) Proportions of saturated fatty acids (SAFA), monounsaturated fatty acids (MUFA), and polyunsaturated fatty acids (PUFA) among the total liver fatty acids in wild type (WT) and *Decr^−/−^* (KO) mice in the fed state and after 24 h of fasting (fasted).

To further analyze the role of DECR disruption in hepatic lipid accumulation, liver lipids were analyzed using positive ion mass spectrometry, as described under Materials and Methods. Under fed conditions, there were no significant differences in the amount or composition of total liver fatty acids between wild type and null mutant mice, as indicated in Figure 6A. The main fatty acid species were palmitic acid (C_16:0_), stearic acid (C_18:0_), oleic acid (C_18:1_), linoleic acid (C_18:2_), and arachidonic acid (C_20:4_). Figure 6C further shows that under fed conditions, the proportion of saturated fatty acids (SAFA), monounsaturated fatty acids (MUFA), and polyunsaturated fatty acids (PUFA) in total liver fatty acids was comparable between wild type and *Decr^−/−^* mice.

Analysis of liver fatty acids after the mice were fasted for 24 h (Figure 6B) indicated that fasting had a minor effect on the lipid content of wild type liver, with an overall increase of 29% in the concentration of fatty acids. The increase in palmitic (C_16:0_) and stearic acid (C_18:0_) concentrations contributed to the increased SAFA concentration, whereas the increased linoleic acid (C_18:2_) concentration contributed to the increased PUFA concentration. There were no significant differences in the amount or composition of total liver fatty acids between wild type and heterozygous mutant mice.

In *Decr^−/−^* mice, however, the overall concentration of fatty acids increased by 108% after fasting. This was in agreement with the lipid accumulation observed in histological sections by Oil red O staining. The most profound changes between fasted wild type and *Decr^−/−^* mice were observed for the levels of palmitoleic acid (C_16:1_), oleic acid, linolenic acid (C_18:3_) and linoleic acid, which were 2.5- to 3.8-fold higher in *Decr^−/−^* mice. In comparison to the fed state, the concentrations of MUFA and PUFA increased by 288% and 254%, respectively, in *Decr^−/−^* mice (Figure 6B). The increased concentrations of MUFA were due to the increase in oleic acid and palmitoleic acid concentrations, whereas the increased PUFA concentrations were due to the increased linoleic acid and linolenic acid concentrations. The effect of fasting was most pronounced for the concentrations of linoleic and linolenic acids, which were increased 5.5-fold and 6.9-fold when compared with the fed state. The concentration of SAFA remained relatively unchanged, although an increase in the concentration of palmitic acid and a decrease in the concentration of stearic acid were observed.

### Acylcarnitine and Dicarboxylic Acid Analysis

Acylcarnitine profiling is commonly used as a biochemical tool to diagnose various inherited metabolic disorders. For example, the initial diagnosis of long-chain fatty acid oxidation disorders is most often performed by analyzing serum or plasma acylcarnitines. Disruption of mitochondrial β-oxidation of long-chain fatty acids leads to intramitochondrial accumulation of acyl-CoA esters, which leak into the blood stream as acylcarnitines after transesterification with carnitine. This means that the serum acylcarnitine profile reflects acyl-CoA esters that accumulate intramitochondrially and pinpoints the site of metabolic block in the oxidation pathway.

**Figure 7:**
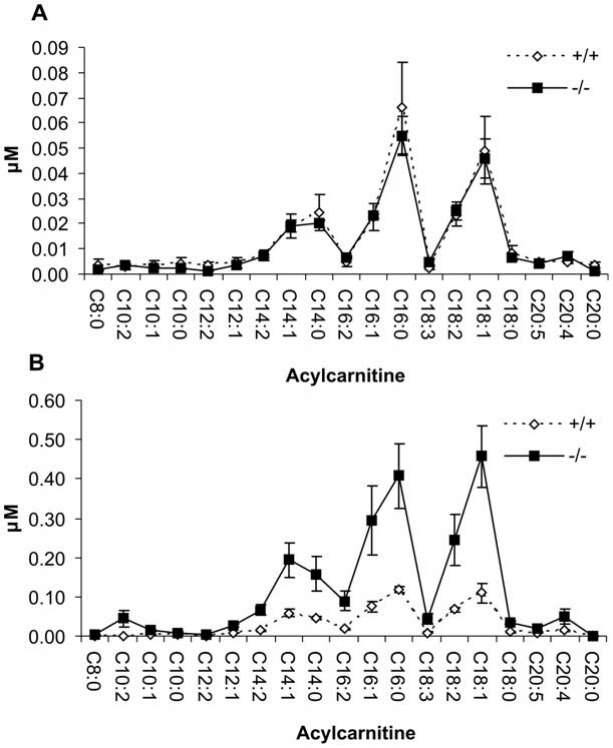
Serum acylcarnitine analysis under normal and fasted conditions. Serum acylcarnitines were analyzed for wild type (solid line) and *Decr^−/−^* mice (dotted line) by mass spectrometry. (A) Serum acylcarnitine profile under normal conditions, as determined from the mass spectral data. (B) To determine the serum acylcarnitine profile under fasted conditions mice were subjected to a 24 h fast prior to serum collection. The concentrations of acylcarnitine are expressed as means±SE of 5–6 mice of each genotype per group.

To study the effect of *Decr* gene disruption on the acylcarnitine profile and whether disruption leads to the secretion of specific acylcarnitine species, serum acylcarnitine profiles were determined for non-fasted and fasted wild type and *Decr^−/−^* mice by mass spectrometry. Under non-fasted conditions, there were no significant differences in the levels of total serum acylcarnitines between wild type and *Decr^−/−^* mice (263±29 nM and 240±16 nM, respectively). In addition, no significant differences were detected in the levels of individual acylcarnitines from C_8_ to C_20_ (Figure 7A). Predominant acylcarnitines in the sera were C_16_ and C_18:1_ acylcarnitines. Fasting increased the total concentration of acylcarnitines by 2-fold in wild type mice (567±32 nM); however, in *Decr^−/−^* mice, a markedly higher 9-fold increase was observed (2150±230 nM). Compared with wild type mice, the levels of all analyzed acylcarnitine species were highly elevated in *Decr^−/−^* mice. The increase was most distinct for the level of decadienoylcarnitine (C_10:2_), the concentration of which was 44-fold higher in the sera of *Decr^−/−^* mice compared with wild type controls (Figure 7B).

To analyze the excretion of dicarboxylic acids, another marker for mitochondrial β-oxidation dysfunction, mice were housed in metabolic cages and urine was collected for 24 h (fed state sample). Subsequently, food was removed and collection was continued for another 24 h (fasted sample). Dicarboxylic acid contents were monitored by means of mass spectrometry. A significant excretion of molecules with masses corresponding C_7:2_, C_8:2_, C_10:2_, C_10:3_, and C_14:3_ dicarboxylic acids was observed in *Decr^−/−^* mice after fasting. The excretion of these dicarboxylic acids, especially C_10:2_ and C_14:3_, which were not detected in the urine of wild type mice, was also observed in the fed state for *Decr^−/−^* mice (Table S2 and Figure S1). C_8:2_ and C_10:2_, the expected products of linoleic acid degradation, reached concentrations of 154 µM and 72 µM, respectively, while C_14:3_, which is expected product of α linolenic acid, was found up to 16 µM. In the urine of wild type mice, their concentrations, with the exception of C_8:2_, were close to or below detection limit (approx. 5 µM).

### Quantitative Real-Time PCR Analysis of Gene Expression

Adaptation to fasting is partially transmitted via altered transcription of genes encoding enzymes that function in multiple pathways. These alterations are directed by transcription factors, coactivators and corepressors that act as sensors of the nutritional status of an organism. To analyze whether disturbances observed in *Decr^−/−^* mice were accompanied by altered expression of genes encoding proteins involved in mitochondrial and extramitochondrial (peroxisomal and microsomal) fatty acids and carbohydrate metabolism, quantitative real-time PCR method was conducted.

**Figure 8:**
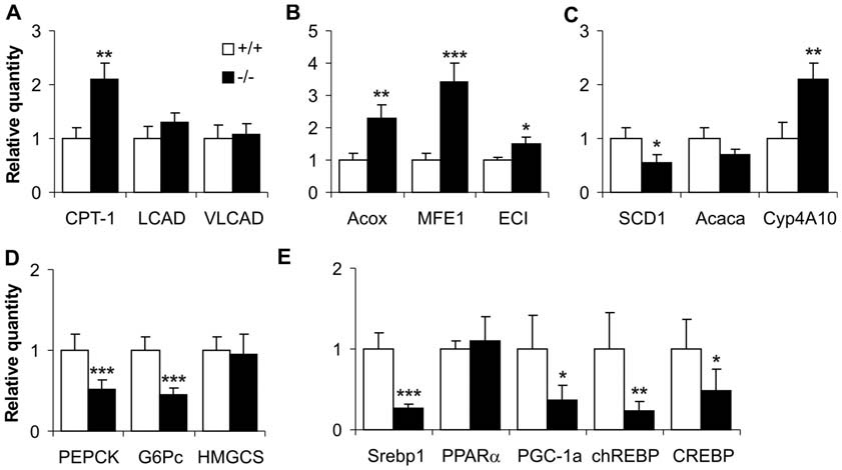
Effect of fasting on hepatic expression levels of genes for mitochondrial and extramitochondrial fatty acid metabolism. Quantitative real-time PCR analysis was used to determine changes in hepatic gene expression in *Decr^−/−^* mice (solid bars) after 24 h of dietary stress compared with wild type mice (open bars). (A) Relative expression levels of genes involved in mitochondrial β-oxidation; CPT-1, LCAD, and VLCAD. (B) Relative expression levels of genes involved in the peroxisomal β-oxidation pathway; Acox, MFE1 and ECI. (C) Relative expression levels of genes involved in fatty acid synthesis, desaturation and microsomal ω-oxidation; Acaca, SCD1 and Cyp4A10, respectively. (D) Relative expression levels of genes involved in the gluconeogenetic pathway and ketone body synthesis; PEPCK, G-6Pase and HMGCS, respectively. (E) Relative expression levels of genes encoding transcriptional factors; PPARα, Srebp1, chREBP, CREB, and the co-activator PGC-1α. For relative quantification of gene expression, the results were normalized using GAPDH as an endogenous control for each sample, and the data obtained for wild type samples were set to 1. Results represent means±SE of 5 mice of each genotype per group. Statistically significant differences in expression levels between wild type and *Decr^−/−^* mice are indicated by asterisks (* p<0.05, ** p<0.01, *** p<0.001).

When the expression levels of several mitochondrial β-oxidation enzymes in the liver were compared between wild type and *Decr^−/−^* mice (Figure 8), a 2-fold increase was observed in the expression level of the rate-limiting enzyme carnitine palmitoyltransferase (CPT-1) in *Decr^−/−^* mice. Expression levels of other studied mitochondrial β-oxidation enzymes, long chain acyl-CoA dehydrogenase (LCAD) and very long chain acyl-CoA dehydrogenase (VLCAD) were comparable, but were slightly increased in *Decr^−/−^* mice (Figure 8A). A greater change was observed in the expression levels of genes associated with peroxisomal β-oxidation, because the expression levels of acyl-CoA oxidase (Acox) and peroxisomal multifunctional enzyme 1 (MFE1) were 2.3- and 3.4-fold higher in *Decr^−/−^* mice, respectively (Figure 8B). In addition, the expression of ECI, which is one of the auxiliary enzymes that functions in the oxidation of polyunsaturated fatty acids with double bonds in odd-numbered positions, was slightly upregulated (1.5-fold). A significant 2.1-fold increase was also observed in the expression level of cytochrome P450 IVA1 (CYP 4A10), which is a key enzyme in microsomal ω-oxidation (Figure 8C). Normally, enzymes involved in fatty acid synthesis and desaturation are downregulated during fasting. The expression level of acetyl-CoA carboxylase (Acaca), which catalyzes the first step in the fatty acid synthesis pathway, was lower in *Decr^−/−^* mice, although it was not significant (Figure 8C). However, the messenger RNA level of the enzyme responsible for synthesis of monounsaturated fatty acids, stearoyl-CoA desaturase 1 (SCD1), was markedly lower in *Decr^−/−^* mice (Figure 8C). During fasting, glucose homeostasis is maintained in part by the production and utilization of ketone bodies and in part by the production of glucose via gluconeogenesis. The hypoglycemic response to fasting prompted us to study the expression of phosphoenoylpyruvate carboxykinase (PEPCK-C) and glucose-6-phosphatase (G6Pase), key enzymes in the gluconeogenesis and glyceroneogenesis pathway, as well as mitochondrial 3-hydroxy-3-methylglutaryl-CoA synthase (HMGCS), an enzyme required for ketone body synthesis (Figure 8D). We found that the levels of PEPCK and G6Pase were decreased (2- and 2.2-times, respectively) in *Decr^−/−^* mice, whereas no differences were detected in the levels of HMGCS. Glucose homeostasis is regulated systemically by hormones such as insulin and glucagon and at the cellular level by energy status. During fasting, glucagon enhances glucose output from the liver via a PKA signal transduction pathway by activating cyclic AMP-responsive element binding protein (CREB), which in turn activates the expression of PPARγ coactivator-1α (PGC-1α) [22]. PGC-1α has been ascribed a central role in controlling the transcription of genes involved in major metabolic pathways in the liver (mitochondrial biogenesis, fatty acid catabolism, oxidative phosphorylation, and mitochondrial biogenesis) through the coactivation of several nuclear receptors and other transcription factors [23]. During fasting, increased PGC-1α levels in the liver induce gluconeogenesis by activating PEPCK and G6Pase promoters through direct interaction with hepatic nuclear factor 4α (HNF4α) and forkhead box transcription factor, FOXO1. Interestingly, we observed significantly decreased expression levels of CREB and PGC-1α (2.1 and 2.8-times, respectively) in *Decr^−/−^* mice compared with wild type mice after fasting (Figure 8E). Although peroxisome proliferator activated receptor α (PPARα) has a central role in the transcriptional control of genes encoding fatty acid oxidation enzymes, further transcription factors are responsible for the regulation of other metabolic pathways (e.g., sterol regulatory element-binding protein (SREBP), which regulates genes involved in lipogenesis, cholesterogenesis, and glucose metabolism and carbohydrate responsive element binding protein (chREBP), which mediates the transcriptional effects of glucose on glycolytic and lipogenic genes). Fasting produced no differences in the expression level of PPARα between wild type and *Decr^−/−^* mice; however, the expression levels of SREBP1 and chREBP in *Decr^−/−^* mice were significantly repressed (0.3 and 0.25 times the level observed in wild type mice after fasting) (Figure 8E).

### Cold Intolerance and Physical Activity

**Figure 9:**
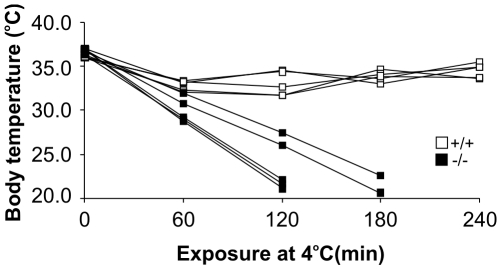
Changes in body temperature during cold exposure with prior fasting. Cold tolerance was tested after 20 h of fasting by exposing individually housed wild type mice (open boxes) and *Decr^−/−^* mice (solid boxes) to a +4°C environment for a maximum of 4 hours or until body temperature dropped below 25°C. Temperature was measured from the shaved mid-dorsal body surface using an infrared thermometer. At each time point, body temperatures are presented as mean values of three measurements from the same individual.

In order to determine the effects of cold stress, mice were fasted for 20 h and exposed to a cold environment (+4°C) for 4 h. *Decr^−/−^* mice exhibited severe cold intolerance during acute cold exposure and the experiment was terminated when body temperature decreased below 25°C. It has been shown that mice with temperatures below 25°C do not recover, and thus this body temperature can be considered terminal without using death as an end-point [24]. After exposure for 2 h, the average body temperature of *Decr^−/−^* mice (n = 5) was 23.4°C compared with 33.0°C for wild type mice, and three of the five *Decr^−/−^* mice demonstrated temperatures that had declined below 25°C (average 21.3°C). The temperatures of the two remaining *Decr^−/−^* mice continued to decline linearly and averaged 21.6°C after 3 h, at which time the experiment was terminated (Figure 9). Shivering was also initially present in *Decr^−/−^* mice but decreased during the experiment and was absent after 2 h, at which time mice became lethargic. In contrast, none of the 5 wild type mice succumbed to cold and, at the end of the 4 h exposure, shivering was clearly present and the average body temperature was 34.5°C. The severely cold intolerant phenotype of *Decr^−/−^* mice was observed only if cold exposure was preceded by fasting (data not shown).

As previously mentioned, *Decr^−/−^* mice showed decreased blood glucose and elevated NEFA concentrations after fasting in comparison to wild type mice. This response was more pronounced in both mouse genotypes when the fasting period was followed by acute cold exposure. When cold exposure was terminated, *Decr^−/−^* mice demonstrated glucose and NEFA concentrations of 3.0±0.2 mmol/l and 1.6±0.02 mmol/l, respectively, whereas the values obtained for wild type mice were 6.3±0.5 mmol/l and 0.77±0.08 mmol/l.

**Figure 10:**
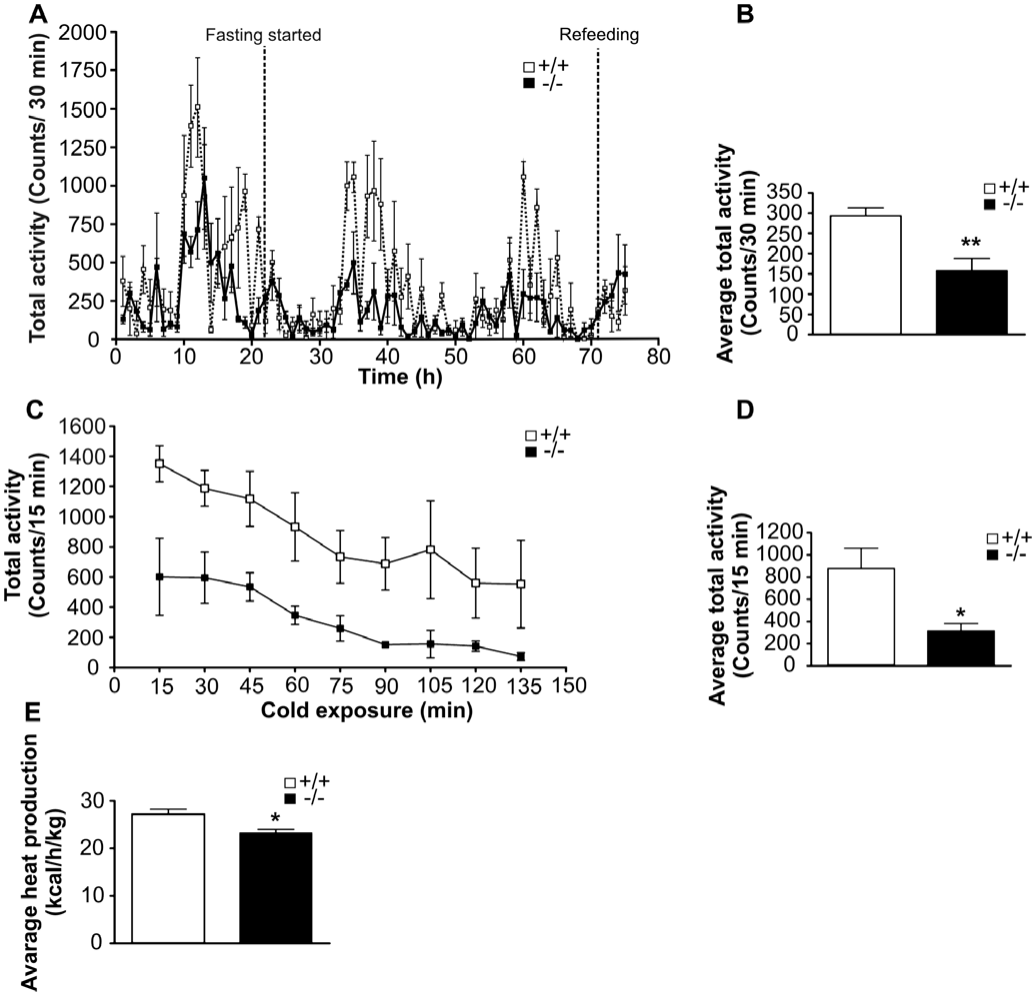
Effect of fasting and cold exposure on physical activity and heat production. To assess physical activity, *Decr^−/−^* mice (solid boxes and bars) and wild type mice (open boxes and bars) were continuously monitored with the LabMaster system. (A) Activity pattern during 48 hours of fasting. Fasting was started at 8 am and continued for 48 hours, with data collected every 30 min. Group means±SEM (n = 4) are shown. (B) Average total activity (counts/30 min) during the 48 h fast. Group means±SEM (n = 4) are shown. (C) Total activity during cold exposure. *Decr^−/−^* and wild type mice were fasted 20 h and then exposed to cold for 2 h (+9.6°C). Total activity was measured continuously and data were collected every 15 minutes. (D) Average total activity (counts/15 min) during cold exposure. Group means±SEM (n = 4) are shown. (E) Average heat production (kcal/h/kg) during cold exposure. Group means±SEM (n = 4) are shown. Student's t-test was used for statistical analysis, and p-values below 0.05 were considered statistically significant. Data for total activity during cold exposure were analyzed by two-way ANOVA followed by Bonferroni's post-test.

To analyze the physical activity of mice during fasting and cold exposure, a LabMaster study was conducted. This study showed that during fasting wild type mice maintained a normal activity pattern, in which activity was highly enhanced during the dark period. When total activity during the 48 h fast was determined, *Decr^−/−^* mice showed significantly lower average activity counts than wild type mice; in particular, their activity was greatly reduced during the dark period (Figure 10A). Average total activity counts were 158±30 counts/30 min and 293±19 counts/30 min for *Decr^−/−^* and wild type mice, respectively (Figure 10B). To assess activity during cold exposure, mice were fasted for 20 h and then exposed to the cold for 2 h. At the beginning of the exposure, *Decr^−/−^* mice displayed significantly lower activity, reflecting the effect of fasting, as shown in Figure 10A and 10B. Upon continuation of cold exposure, the activity of both mouse groups decreased in a similar manner. However, at the end of the 2 h exposure period, the activity of *Decr^−/−^* mice had decreased to close to zero, whereas wild type mice maintained a reasonable amount of activity (Figure 10C). Average total activity counts for wild type and *Decr^−/−^* mice during cold exposure were 878±183 counts/15 min and 317±65 counts/15 min, respectively (Figure 10D). Average heat production during cold exposure was also measured, and *Decr^−/−^* mice showed a slight but significant decrease in heat production. The average heat production of wild type and *Decr^−/−^* mice was 27.1±1.2 kcal/h/kg and 23.2±0.8 kcal/h/kg, respectively (Figure 10E).

## Discussion

The results presented herein indicate that mitochondrial 2,4-dienoyl-CoA reductase activity in mice is indispensable for the complete oxidation of (poly)unsaturated fatty acids and for adaptation to metabolic stress. *Decr^−/−^* mice exhibited hypoglycemia during fasting, with concomitant accumulation of metabolites of unsaturated fatty acids in the liver, sera, and urine. Furthermore, a predisposition to cold intolerance and a reduction in diurnal activity during metabolic stress were observed.

Insufficient adaptation to metabolic stress in *Decr^−/−^* mice is exemplified by the development of microvesicular hepatic steatosis after as little as 24 h of fasting. However, levels of serum alanine aminotransferase, alkaline phosphatase, and glutamyl transferase were similar in both *Decr^−/−^* and wild type animals, suggesting that null mutant mice did not develop liver cell membrane injury during the observation period.

In the *Decr^−/−^* mice, analysis of fatty acid composition in total liver lipids revealed a specific accumulation of mono- and polyunsaturated fatty acids, with oleic and linoleic acids being the dominant species. This can be explained in terms of their impaired β-oxidation, resulting in their channeling toward esterification and leading ultimately to hepatic steatosis. The fact that saturated fatty acids, such as palmitic and stearic acids, which, together with mono- and polyunsaturated fatty acids (especially oleic and linoleic acid), are the main components of triacylglycerols in adipose tissue [25], did not accumulate suggest that they were effectively metabolized. The proceeding β-oxidation of saturated fatty acids explains the carbon source for ketogenesis, which was found to be similar in wild type and *Decr^−/−^* mice.

A prominent feature of *Decr^−/−^* mice is the hypoglycemic response to fasting, which seems not to be related to differences in glycogen or amino acid metabolism, but is associated with altered transcriptional control mechanisms in the gluconeogenesis pathway. Indeed, there were no significant differences in liver and muscle glycogen content in wild type and *Decr^−/−^* mice, either in the fed or in the fasted state. This finding suggested that the observed hypoglycemia was not due to a failure in glycogen metabolism. The amino acid profile and levels in the sera of wild type and *Decr^−/−^* mice after fasting were similar (Table S1) giving no metabolic implications of specific pathological states in *Decr^−/−^* mice. This indicates that the availability of substrates for gluconeogenesis, in terms of gluconeogenic amino acids, was not the limiting factor when hypoglycemia developed in *Decr^−/−^* mice. Furthermore, the levels of glucagon and insulin in the fed state and during fasting showed no marked differences between wild type and *Decr^−/−^* mice, indicating that adaptation to fasting was not affected at the hormonal level.

**Figure 11:**
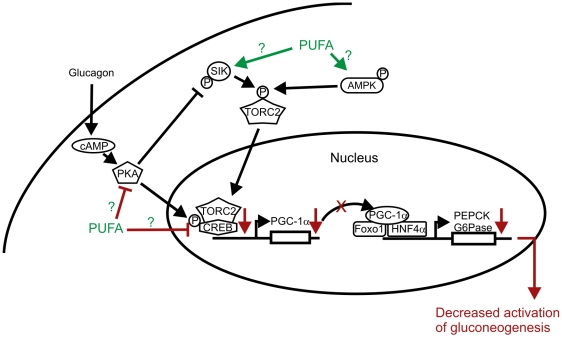
PUFA and activation of gluconeogenesis. Simplified schematic presentation of the identified kinase cascades regulating gluconeogenesis. The activation cascade leads to phosphorylation of CREB (cAMP-responsive element binding protein), which, together with TORC2 (transducer of regulated CREB activity 2), drives the expression of coactivator PGC-1α. Transcription of the key gluconeogenic enzymes PEPCK (phosphoenoylpyruvate carboxykinase) and G6Pase (glucose-6-phosphatase) is induced when PGC-1α associates with HNF4α (hepatic nuclear factor 4α) and FOXO1 (forkhead box transcription factor). Nuclear translocation of TORC2, which is needed for activation of the gluconeogenic program, is controlled by phosphorylation by activated AMPK (AMP–activated protein kinase) and SIK (salt-inducible kinase). Observed changes in *Decr^−/−^* mice are indicated with red arrows. Possible targets of accumulated PUFA or their derivatives are also indicated.

PEPCK and G6Pase are regarded, under *in vivo* conditions, as unidirectional enzymes that, among other factors, control the gluconeogenetic flux and show increased activity in the fasted state. The detected low PEPCK and G6Pase mRNA levels can explain, in part, the hypoglycemia observed in *Decr^−/−^* mice via contributing to the decreased flux through the gluconeogenetic pathway in the liver. Decreased mRNA levels of coactivator PGC-1α and its inducer CREB, which together drive the expression of PEPCK and G6Pase via HNF4α and FOXO1, suggest that signaling pathways leading to activation of gluconeogenesis during fasting are compromised in *Decr^−/−^* mice. However, which of the component(s) upstream of PGC-1α or CREB, especially TORC2 (transducer of regulated CREB activity 2), energy sensing kinase AMPK, and salt-inducible kinase (SIK), are affected and whether this effect is mediated by certain accumulated PUFA species will be evaluated in further studies (Figure 11). Analysis of the expression levels, activity and phosphorylation status of these factors in *Decr^−/−^* mice could elucidate the regulatory link between gluconeogenesis and the disrupted breakdown of unsaturated fatty acids.

The expression of several genes, including CPT-1, Acox, and CYP4A1, has been shown to be upregulated by polyunsaturated fatty acids [26],[27]. In addition, unsaturated fatty acids were previously shown to impact different transcription factors that play a pivotal role in lipid and carbohydrate metabolism. Thus, the accumulated unsaturated fatty acids and their derivatives in *Decr^−/−^* mice can act as regulators of gene expression by functioning as ligands for nuclear receptors, such as PPARα, which is the major PPAR subtype present in hepatocytes and is involved in regulating genes involved in lipid and carbohydrate metabolism [28],[29]. In accordance, the expression of the PPARα target genes CPT-1, Acox, and CYP4A1 was markedly higher after fasting in *Decr^−/−^* mice compared with wild type mice. Unsaturated fatty acids can also affect gene expression via SREBP1, a factor considered a key regulator of triacylglycerol and fatty acid synthesis [30]. Although it is believed that they function mainly by inhibiting the maturation of lipogenic SREBP1 [31],[32], they have been shown to decrease hepatic SREBP1 mRNA [33]. In line with these observations, the mRNA level of SREBP1 was reduced 3-times in *Decr^−/−^* compared with wild type mice after fasting, and the levels of SCD1 and Acaca mRNAs, which are mediated in part by SREBP1, were decreased as well. PUFA has recently been shown to have a suppressive effect on lipogenic and glycolytic gene expression through chREBP [34]. Namely, PUFA ablates chREBP translocation from the cytosol to nucleus and accelerates chREBP mRNA decay [35],[36]. Of note, the *Decr^−/−^* mice displayed significantly decreased (4-times lower) levels of chREBP mRNA compared with wild type animals after fasting, likely reflecting the increased chREBP mRNA decay mediated by accumulated PUFA.

In our experimental setting of acute cold exposure (4 h) without prior acclimatization, it is unlikely that nonshivering thermogenesis plays any major role in maintaining mouse body temperature. The decreased heat production observed in *Decr^−/−^* mice in the fasted state can be explained by reduced shivering [37]. Reduced shivering can in turn be explained by the reliance of fast-twitching muscle fibers on glucose, which is depleted in *Decr^−/−^* mice under these conditions. Wild type mice display a diurnal activity pattern with higher physical activity during the dark phase, and this pattern is preserved during fasting [38]. This increased dark phase activity, however, was greatly diminished in fasted *Decr^−/−^* mice. Of note, no differences in thermoregulation were observed between wild type and *Decr^−/−^* mice in the fed state. Because both animal groups generated ketone bodies, which can be utilized as fuel in muscle tissue, the link between simultaneous hypoglycemia and the incapability to maintain body temperature in *Decr^−/−^* mice remains an intriguing open question.

By inspecting the acylcarnitine profile of the sera of *Decr^−/−^* mice, one can deduce which paths are used by unsaturated fatty acids during their degradation. The mitochondrial β-oxidation of unsaturated fatty acids with preexisting double bonds at even-numbered positions, such as petroselinic acid (C_18:1_, Δ^12^), is predicted to halt in *Decr^−/−^* mice during the fourth turn of the β-oxidation cycle after *cis*-4-decenoyl-CoA is dehydrogenized to *trans*-2-*cis*-4-decadienoyl-CoA (Figure 1, left section). If the double bond is in an odd-numbered position, such as in oleic acid (C_18:1_, Δ^9^), mitochondrial oxidation can proceed to completion via the ECI-dependent route (Figure 1, middle section) or can be halted during the third β-oxidation cycle at the level of *trans*-2-*cis*-4-tetradecenoyl-CoA, which can be generated from the 2,5-tetradecenoyl-CoA intermediate (Figure 1, right section) by the combined activity of ECI and DECI [39]. For polyunsaturated fatty acids having double bonds at odd- and even-numbered positions, such as in linoleic acid (C_18:2_, Δ^9,12^) , the lack of DECR activity results in blocking of the β-oxidation of these fatty acids either when intermediates of odd-numbered bonds are routed to the reductase-dependent pathway or when the even-numbered double bond reaches the Δ^4^ position during acyl chain shortening via β-oxidation.

The distinctive accumulation of *trans*-2,*cis*-4-decadienoylcarnitine (C_10:2_) in the sera of *Decr^−/−^* mice can be derived from the incomplete oxidation of unsaturated fatty acids of the ω-6 series. Among them, linoleic acid is the most abundant species in the animal body, whereas others, such as petroselinic acid, are substantially less frequent. Because no intermediates of the reductase-dependent pathway for unsaturated fatty acids with double bonds at odd-numbered positions were observed, the data suggest that the major route for β-oxidation of these fatty acids proceeds via the isomerase-dependent pathway *in vivo*. If the reductase-dependent pathway were preferred, it would lead to accumulation of tetradecatrienoylcarnitine (C_14:3_, Δ^2,4,8^) and tetradecadienoylcarnitine (C_14:2_, Δ^2,4^) from the incomplete oxidation of linoleic and oleic acids, respectively. In line with this notion, the accumulation of C_14:2_ metabolites (e.g., intermediates of linoleic acid β-oxidation via the reductase-dependent route) was not observed. Although total acylcarnitine concentrations in *Decr^−/−^* mice after fasting were approximately 4-fold higher compared with those of wild type, the profile of acylcarnitines showed no distinctively accumulating species other than *trans*-2,*cis*-4-decadienoylcarnitine. This finding agrees with studies examining the breakdown of 2,5-octadienoyl-CoA in isolated rat liver mitochondria, namely, most of the test substrate (80%) in this system was metabolized via the ECI-dependent pathway [40].

The accumulated unsaturated fatty acids observed in *Decr^−/−^* mice could also act as substrates in alternative oxidation pathways and processes. The microsomal fatty acid ω–hydroxylation can, together with peroxisomal β-oxidation pathway, provide an alternative route to prevent the accumulation of fatty acids or their derivatives in hepatocytes during times of increased lipolysis [41]. Both ω-oxidation and peroxisomal β-oxidation are induced in *Decr^−/−^* mice, as indicated by the enhanced expression of microsomal Cyp4A10 and peroxisomal Acox and MFE1. Consequently, processing of accumulated unsaturated intermediates by microsomes and peroxisomes in *Decr^−/−^* mice can explain the observed excretion of medium chain unsaturated dicarboxylic acids into the urine.

Many of the clinical characteristics observed in human patients suffering from fatty acid oxidation disorders can be reproduced in mice, as shown for mouse models of SCAD, MCAD, LCAD, and VLCAD deficiencies [19],[20],[42],[43]. The stress-induced hypoglycemia observed in VLCAD-deficient mice was recently shown to be linked to impaired gluconeogenesis, but whether impairment is caused by inhibition of certain enzymes in the pathway or due to alterations at the level of transcription remains unknown [44]. The mouse model for MCAD does not suffer from hypoglycemia, although it is cold intolerant and displays lower blood glucose. A recent study indicated that severe metabolic stress leads to specific changes in carbohydrate management in MCAD-deficient mice [45]. Null mutant mouse models for defects in fatty acid breakdown frequently display more severe phenotypes than the corresponding deficiencies in humans, although this is not the case for Decr. To date, only a single clinical case presenting the DECR deficiency has been published [15]. Metabolic studies revealed abnormal plasma and urine acylcarnitine profiles, with the dominant species corresponding to decadienoylcarnitine, hypocarnitinemia and hyperlysinemia. Despite carnitine supplementation and a change in dietary fat to mainly medium-chain triacylglycerols, the patient died at the age of four months. DECR activity measured in postmortem liver and muscle samples was found to be decreased to 40% of the normal activity in liver and 17% of the normal activity found in muscle, as measured using *trans*-2-*cis*-4-decadienoyl-CoA as a substrate [15]. Consistent with the characteristics of the clinical case, the same dominant carnitine species was observed in *Decr^−/−^* mice, although hyperlysinemia was not observed. An open question, therefore, remains as to whether the primary cause of patient death was due to DECR deficiency or whether the patient suffered another disease that remained undiagnosed.

One mouse model is known in which mitochondrial β-oxidation of unsaturated fatty acids is halted at the level of their *cis*- or *trans*-3-enoyl-CoA intermediates due to disruption of ECI [16]. Similar to *Decr^−/−^* mice, *Eci^−/−^* mice are asymptomatic under fed conditions but upon fasting, accumulate unsaturated acyl groups in ester lipids and develop hepatic steatosis. ECI deficiency also led to dicarboxylic aciduria, with an accumulation of medium chain unsaturated dicarboxylic acids. However, whether *Eci^−/−^* mice have a hypoglycemic response to fasting or show cold intolerance, as observed for *Decr^−/−^* mice, was not reported. In addition, production of ketone bodies was not reported in *Eci^−/−^* mice. Thus, a lack of information prevents a thorough comparison of *Eci^−/−^* and *Decr^−/−^* mice.

In the present study, we examined the physiological consequences of disruption of the mitochondrial β-oxidation of unsaturated fatty acids at the level of 2,4-dienoyl-CoA reductase. This mouse model provides for the first time a hint that the breakdown of PUFA is essential for switching on gluconeogenesis during fasting. *Decr^−/−^* mice may serve as a model for studying the mechanism responsible for idiopathic hypoglycemia with unimpaired ketogenesis in humans. Analysis of the expression profile of selected transcription factors and cofactors revealed the involvement of CREB and PGC1α upstream of the reduced expression of PEPCK and G6Pase in *Decr^−/−^* mice. Among accumulated acylcarnitines in the sera, *trans*-2,*cis*-4-decadienoylcarnitine (C_10:2_), which is a potential novel metabolic marker for screening patients with inborn errors in polyunsaturated fatty acids breakdown, was found.

## Materials and Methods

### Generation of *Decr* Knockout Mice

The genomic clone BACM:109-18E (from 129/SvJ strain) corresponding to the mouse *Decr* locus was obtained from Genome Systems (St Louis, MO, USA). A 3.3 kb *Eco*RI–*Hind*III fragment upstream of the first exon was cloned into a Bluescript vector modified with *Sal*I and *Cla*I sites flanking the polylinker. A 4.4 kb *Sma*I–*Eco*RV fragment from the first intron was cloned into a Bluescript vector modified with *Asc*I and *Pac*I sites flanking the polylinker. For the replacement vector, *Sal*I–*Cla*I and *Asc*I–*Pac*I cleaved fragments were ligated to the corresponding sites of the pPGKneo/TK-2 vector (Figure 2A), where they flanked the PGKneo cassette. The neomycin resistance (*neo*) and thymidine kinase (TK) genes were used for positive and negative selection, respectively.

Linearized replacement vector was electroporated into RW4 embryonic stem (ES) cells (129/SvJ, *Tyr^ch^p/Tyr^c^p*) that were subsequently grown under G418 and ganciclovir selection. Correctly targeted ES cell clones were identified by Southern analysis of genomic DNA, for which the *Bam*HI restriction fragment length polymorphism created by homologous integration was identified by a 5′-probe upstream of the targeted locus (Figure 2B). Germline chimeric mice were produced by microinjecting ES cells from positive clones into C57BL/6 blastocysts at the Biocenter Oulu Transgenic core facility. Genotyping was performed by PCR analysis of tail DNA samples using forward primers for the wild type allele (5′- TGC GTT CTT TGC TGG GGT GTC C-3′) and for the mutated allele (5′-CTC GAG AT C CAC TAG TTC TAG CC -3′) and a reverse primer for both alleles (5′-CAA ATG AAA GTT CCC TTG TGG AG-3′) (Figure 2C). The size of the amplified products was 382 bp and 280 bp for the wild type and the mutated allele, respectively.

### Animal Studies

Four- to seven-months-old mice were used in all experiments. DECR null mice were backcrossed 9 times onto the C57BL/6 background and C57BL/6 mice were used as wild type controls. Mice were housed in an animal room with a 12-hour lighting period (07:00–19:00) and given unrestricted access to water and standard chow. For fasting experiments, the mice were housed individually and food was withdrawn for 6 to 48 hours; water was provided *ad libitum*. Cold tolerance was tested by exposing individually housed fasted (20 h) or non-fasted mice to +4°C for a maximum of 4 hours or until their body temperature dropped below 25°C. Temperature was measured from the shaved mid-dorsal body surface using a ThermoScan thermometer (PRO 4000, Braun, Kronberg, Germany), as described earlier [46].

Oxygen consumption, CO_2_ production, energy expenditure, determination of food and water intake, and activity (total activity, ambulatory and fine movement and rearing) were measured simultaneously and continuously in housing cages utilizing an indirect open circuit calorimetry system with a dual array of infrared photo beams (LabMaster, TSE Systems, Bad Homburg, Germany). Before the experiments were carried out, mice were acclimated to their new environment in training cages similar to the actual experimental cages for seven days. During the experiment, mice were individually housed in Plexiglas home cages, fresh air was supplied at a constant flow of 0.33 l/min, and O_2_ consumption and CO_2_ production were measured and compared with room air values. Data were collected every 30 min for 72 h. The respiratory exchange ratio was calculated by dividing the volume of CO_2_ production (VCO_2_) by the volume of oxygen consumption (VO_2_), and energy expenditure (heat production) was calculated using the software provided with the instrument. For fasting studies, mice were similarly acclimated to the experimental conditions and their baseline (mice fed *ad libitum*) was analyzed over 24 hours. Food was then removed at 8 am and fasting was continued for 48 hours; the mice were analyzed continuously, as described above. For the cold exposure study in the LabMaster system, mice were fasted overnight (20 hours) prior to the experiments. Plexiglas cages were placed in a refrigerated cold cabinet (Helkama, Finland) with a controlled temperature (average temperature of 9.6°C) for 2 hours. Data were collected every 15 minutes and analyzed using Microsoft Excel and GraphPad Prism version 4.03.

All animals were handled in strict accordance with good animal practice and their use in the present study was approved by the University of Oulu committee of animal experimentation. When provided, the values represent means±S.E.

### Immunoblotting and Activity Measurements

To isolate mitochondria from heart and skeletal muscle tissues, 200–500 mg of tissue was cut into small pieces in 10 volumes (w/v) of isolation buffer (100 mM KCl, 50 mM HEPES, pH 7.4, 5 mM MgCl_2_, 1 mM EDTA). The solution was replaced with 10 volumes of isolation buffer containing 2 mg/ml of the bacterial protease Nagarse (Sigma, St. Louis, MO, USA) and the tissue samples were incubated on ice for 5 min. Samples were washed with 10 volumes of isolation buffer and subsequently homogenized in 10 volumes of isolation buffer containing 2 mM ATP with a motorized glass-teflon homogenizer. The suspension was centrifuged at 3000×g for 4 min and the resulting supernatant was further centrifuged at 17000×g for 10 min to pellet the mitochondria. The resulting pellet was suspended in 1.4 volumes of suspension buffer (10 mM Tris-Cl, pH 7.8, 250 mM sucrose, 0.2 mM EDTA). To produce the mitochondrial homogenate from liver tissue, 500 mg of tissue was homogenized in 10 volumes of isolation buffer, followed by centrifugation as described above and resuspension in suspension buffer. To minimize peroxisomal contamination, mitochondrial purification was completed by isopycnic density gradient ultracentrifugation on a self-generating Percoll (Sigma) gradient, as previously described [17]. For immunoblotting, samples containing 20 µg of protein were transferred to a nitrocellulose membrane after SDS-PAGE and detected using polyclonal antibody against rat 2,4-dienoyl-CoA reductase [47] as the primary antibody and goat anti-rabbit IgG horseradish peroxidase conjugate (Bio-Rad Laboratories, Hercules, CA, USA) as the secondary antibody, followed by ECL Western Blotting Detection Reagents (Amersham Biosciences, Piscataway, NJ, USA).

The 2,4-Dienoyl-CoA reductase activity was assayed in mitochondrial extracts by spectrophotometric measurement of NADPH consumption at 22°C using 60 µM 2,4-hexadienoyl-CoA as substrate, as previously described [10].

### Blood Chemistry

Blood samples were collected from anesthetized mice by orbital bleeding in Multivette collection tubes (Sarsted, Nümbrecht, Germany) and serum was separated by centrifugation after 15 min. After being bled, mice were sacrificed by cervical dislocation and tissues were weighed and collected for further analysis. Serum cholesterol, triacylglycerols, albumin, alkaline phosphatase, alanine aminotransferase, glutamyl transferase, β-hydroxybutyrate and amino acids were analyzed by the clinical laboratory of the University Hospital of Oulu, Finland. Serum glucose (Glucose, Wako Chemicals, Neuss, Germany) and free fatty acids (NEFA C, Wako Chemicals) were determined by enzymatic colorimetric methods. Insulin levels were measured with the insulin ELISA kit (Chrystal Chem Inc., IL, USA) using mouse insulin as a standard. Glucagon was determined using the glucagon RIA kit (Linco Research Inc, MO, USA).

### Glycogen Analysis

Glycogen content was determined by the phenol–sulfuric acid method modified from Lo *et al*. [48]. Portions of frozen liver and muscle (50–90 mg) were weighed and placed in test tubes containing 1.0 ml of 5 M KOH solution saturated with sodium sulfate. The tubes were placed in a boiling water bath for 30 min to obtain a homogenous solution. Tubes were cooled on ice for 5 min, and glycogen was precipitated by the addition of 1 ml of 95% ethanol and incubation on ice for 30 min. Samples were centrifuged at 840×g for 30 min, after which the supernatants were removed and the precipitates were dissolved in 3 ml of distilled water. Aliquots of the glycogen solutions, including standards, were made up to 1 ml in water. One milliliter of 5% phenol solution and 5 ml of a concentrated sulfuric acid solution were added in rapid succession to each tube. The tubes were allowed to stand for 10 min at room temperature, their contents thoroughly mixed, and the tubes were further incubated in the water bath (25°C) for 10 min, followed by measurement of their absorbances at 490 nm.

### Mass Spectrometric Analysis of Total Liver Fatty Acids and Serum Acylcarnitines

Mass spectral analyses were performed using an APEX II FTICR-MS equipped with an Apollo ESI source (Bruker Daltonics, Bremen, Germany) in the positive ion mode. To assess the level of hepatic fatty acids, frozen liver samples were homogenized in H_2_O (1∶20, w/v) with a Potter homogenizer. Ten microliters of homogenate was added to Eppendorf tubes in which 20 µl of 10 mM pentadecanoic acid had been evaporated at room temperature. Then, 180 µl of CH_3_CN and 20 µl of 5 M HCl was added to the tube and heated for 1 h at 95°C. After being cooled to room temperature, 190 µl of 1 M KOH was added and the tubes were again heated for 1 h at 95°C. After being cooled to room temperature, 100 µl of 5 M HCl was added to the tubes, and hydrolyzed fatty acids were extracted 2 times with 0.5 ml of hexane. Combined extracts were evaporated at room temperature under a stream of nitrogen. The residue was resuspended in 50 µl of 5% oxalylchloride in CH_3_CN (v/v) and heated for 5 min at 50°C. The solution was evaporated at room temperature under a stream of nitrogen. Then, 50 µl of 5% dimethylaminoethanol in CH_3_CN (v/v) was added and, after 5 min, the solvent was evaporated at room temperature under a stream of nitrogen. For MS measurements, the residue was resuspended in 500 µl of MeOH/H_2_O/Acetic acid [49.5/49.5/1; (v/v/v)] and further diluted 1∶50 in the same solvent.

To determine serum acylcarnitines, 10 µl of 10 µM dodecanoyl-carnitine in H_2_O and 100 µl of serum were added to the CHromabond C-8 matrix (Macherey-Nagel, Düren, Germany) in an Eppendorf tube and mixed. The supernatant was discarded after centrifugation and the residue was washed twice with 0.5 ml of H_2_O. Lipids bound to the C-8 material (including the acyl carnitines) were extracted twice with 0.4 ml of CHCl_3_/MeOH (7/2; v/v). Combined extracts were evaporated under a stream of nitrogen at 65°C. The C-8 material was further extracted with 0.6 ml of CHCl_3_/MeOH (7/2; v/v) and the liquid phase was combined with previously evaporated extracts. After being mixed, extracts were centrifuged (16000×g for 1 min) and 550 µl of the liquid phase was carefully transferred to a new tube. The combined extracts were evaporated under a stream of nitrogen at 65°C. The dried pellets (containing lipids including acylcarnitines) were resuspended in 100 µl of a solution containing 1 M acetylchloride in MeOH and heated to 65°C for 15 min. Neutral lipids and acyl-methyl esters were extracted twice with 0.5 ml of hexane. The upper phase (containing the methyl esters) was discarded and the lower phase (containing the acylcarnitines) was evaporated at room temperature under a stream of nitrogen. Shortly before the MS measurements, the residues were resuspended in 30 µl of MeOH/2%AcOH (50/50; v/v). Mass spectral data were recorded in positive ion mode by the accumulation of 256 scans at 256K resolution.

### Determination of the Dicarboxylic Acid Content of Urine

Creatinine content was determined in the urine samples using the method of Popper et al. [49]. The samples were diluted with phosphate buffered saline to a creatinine concentration of 1 mM. Methanol solutions (1 mM) of various dicarboxylic acids were used as standards. Each sample was measured twice: once with standards containing odd-numbered carbon atoms (C_5_-, C_7_-, C_9_-, and C_11_-dicarboxylic acid) and tetradecanedioic (C_14_) acid as a reference and once with the standard mixture containing even-numbered carbon atoms (C_4_-, C_6_-, C_8_-, and C_10_-dicarboxylic acid) and tetradecanedioic acid as a reference. When tested separately the concentration of tetradecanedioic in the urine samples of mice was below the detection limit of the assay used, allowing the use of this acid as a reference (see below).

For quantitative determination of the dicarboxylic acids, 10 µl of standard solution was evaporated under a stream of nitrogen in eppendorf tubes, followed by the addition of 200 µl of the normalized urine sample. Non-carboxylic acid lipids were removed by extraction with three times 0.5 ml diethylether after increasing the pH with 350 µl 0.3 M NaOH. Carboxylic acids were extracted three times with 0.5 ml diethylether after the pH was decreased with 50 µl 6 M HCl. The acid extract was evaporated in a stream of nitrogen at room temperature. The residue was dissolved in 100 µl of 20 mM N,N′-carbonyldiimidazole in acetonitrile and incubated at room temperature for 1 h. Thirty microliters of a solution containing 21 µl of acetonitrile, 3 µl of acetic acid, and 6 µl of phenylethylamine was added, and the samples were incubated for 1 h at room temperature. Finally, the entire sample was evaporated under a stream of nitrogen at 60°C. To desalt the sample, 25 µl of RP-18 (bed-vol.) (ICN, Eschwege, Germany) was prewashed with 0.5 ml methanol, resuspended in 0.5 ml of 0.1% TFA in acetonitrile (9/1, v/v), and added to the evaporated assay-mixture, followed by mixing and centrifugation. The supernatant was then discarded and the residue was again washed with 0.5 ml of 0.1% TFA in acetonitrile (9/1, v/v), and extracted with 200 µl methanol. For the MS measurements, 50 µl of the extract was diluted in 50 µl of MeOH/H_2_O/AcOH (49.5/49.5/1, v/v/v). Mass spectral data were recorded by accumulation of 256 scans at 256K resolution in positive mode. The intensities of the signals in the MS spectras were determined using the Xmass software (Bruker, Bremen) in order to compare the concentrations of dicarboxylic acid amides in the mouse urines. The intensity of the peak corresponding to the mass of tetradecanedioic acid amide was used as reference for the calculation of the concentrations of other metabolites. The intensity of the signals corresponding to the masses of the other standard substances was used as control for the similar behavior of dicarboxylic acid compounds with different chain length.

### Real-Time Quantitative PCR

For real-time quantitative PCR analysis of *Decr* and several other genes involved in fatty acid metabolism, cDNA was produced using a First Strand cDNA Synthesis Kit (MBI Fermentas, Heidelberg, Germany) from total RNA isolated from mouse liver samples with the RNeasy Mini Kit (Qiagen, Hilden, Germany). Real-time quantitative PCR was performed with a 7500 Real Time PCR System (Applied Biosystems, Foster City, CA, USA) using fluorogenic probe-based TaqMan chemistry with Taqman Universal PCR Master Mix (Applied Biosystems) according to the manufacturer's instructions. Primers and 5′ FAM-labeled probes were designed using Primer Express software (Applied Biosystems) and the sequences available in Genbank and were purchased from Sigma-Genosys (Sigma-Genosys, Haverhill, UK). For relative quantification of gene expression, the results were normalized with GAPDH as an endogenous control for each sample and analyzed using 7500 System Software (Applied Biosystems).

### Histological Analysis

For light microscopy analysis, samples from various tissues were fixed in 4% paraformaldehyde in potassium phosphate buffer (100 mM, pH 7.4), embedded in paraffin, sectioned and stained with hematoxylin and eosin. To stain the lipids, tissue samples were embedded in O.C.T. compound (Tissue-Tek, Zoeterwoude, Netherlands) and frozen in liquid nitrogen. Ten micrometer cryosections were cut from frozen samples with a Reichert-Jung 2800 Frigocut cryomicrotome and stained with Oil red O and hematoxylin using standard methods.

## Supporting Information

Figure S1 Urine dicarboxylic acid profiles. Urine of the wild type (dotted line) and *Decr−/−* mice (solid line) was collected for 24 h (fed sample) and collection was continued for 24 h after food removal (fasted sample). Pooled samples (5 mice/group) were normalized to urine creatinine and analyzed with mass spectrometry using tetradecanedioic acid (C14:0) as a reference. (A) Urine dicarboxylic acid profile without fasting. (B) Urine dicarboxylic acid profile after fasting.
(0.18 MB TIF)

Table S1 Amino acid analysis from sera of fasted mice. Sera of the wild type and *Decr−/−* mice were collected after 24 h fast. Amino acids were analyzed by the clinical laboratory of the University Hospital of Oulu, Finland. The concentrations are expressed as means±SEM of 4 mice of each genotype per group.
(0.07 MB PDF)

Table S2 Urine dicarboxylic acid analysis. Urine of the wild type and *Decr−/−* mice (5 mice/group) was collected for 24 h (fed sample) and collection was continued for 24 h after food removal (fasted sample). Pooled samples were normalized to urine creatinine and analyzed with mass spectrometry using tetradecanedioic acid (C14:0) as a reference.
(0.06 MB PDF)
