# Supplementary figures and images for: Mitochondrial 2,4-dienoyl-CoA Reductase Deficiency in Mice Results in Severe Hypoglycemia with Stress Intolerance and Unimpaired Ketogenesis
Source: PLoS Genet. 2009 Jul 3;5(7):e1000543. doi: 10.1371/journal.pgen.1000543 (PMC2697383; doi:10.1371/journal.pgen.1000543)

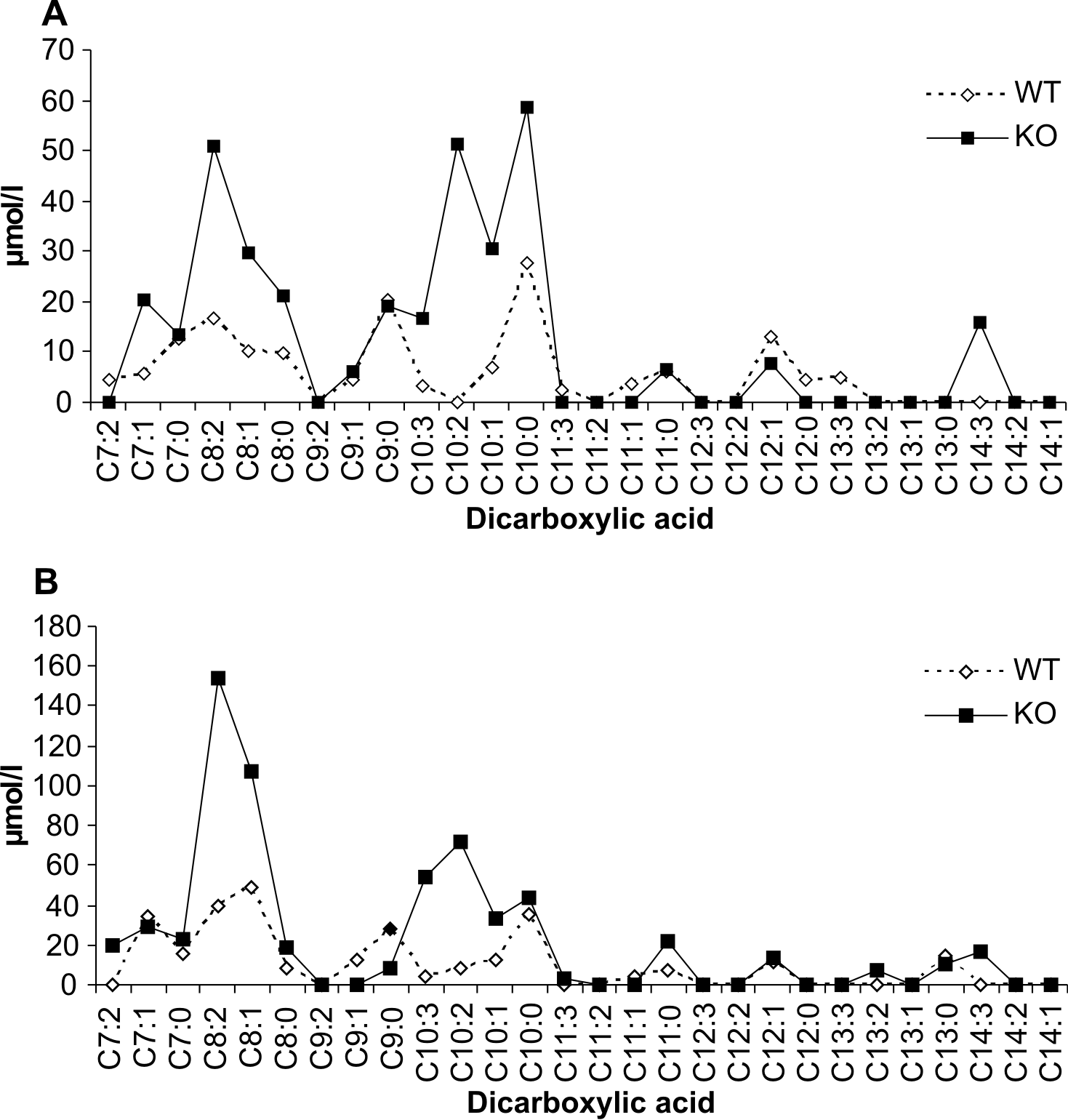

Supplement: Figure S1 — Urine dicarboxylic acid profiles. Urine of the wild type (dotted line) and Decr−/− mice (solid line) was collected for 24 h (fed sample) and collection was continued for 24 h after food removal (fasted sample). Pooled samples (5 mice/group) were normalized to urine creatinine and analyzed with mass spectrometry using tetradecanedioic acid (C14:0) as a reference. (A) Urine dicarboxylic acid profile without fasting. (B) Urine dicarboxylic acid profile after fasting. (0.18 MB TIF) [file pgen.1000543.s001.tif]
