# Supplementary material for: Mitochondrial 2,4-dienoyl-CoA Reductase Deficiency in Mice Results in Severe Hypoglycemia with Stress Intolerance and Unimpaired Ketogenesis
Source: PLoS Genet. 2009 Jul 3;5(7):e1000543. doi: 10.1371/journal.pgen.1000543 (PMC2697383; doi:10.1371/journal.pgen.1000543)
Supplement: Table S1 — Amino acid analysis from sera of fasted mice. Sera of the wild type and Decr−/− mice were collected after 24 h fast. Amino acids were analyzed by the clinical laboratory of the University Hospital of Oulu, Finland. The concentrations are expressed as means±SEM of 4 mice of each genotype per group. (0.07 MB PDF) [file pgen.1000543.s002.pdf]

TABLE S1. Amino acid analysis from sera of fasted mice.

	WT	KO
	Quantity (μmol/l)	
Aspartic acid	4.7 ± 1.0	4.0 ± 0.6
Threonine	131.0 ± 15.3	103.0 ± 3.8
Serine	84.7 ± 8.4	79.6 ± 11.5
Phosphoserine	5.6 ± 0.7	7.4 ± 1.1
Asparagine	35.8 ± 2.2	37.1 ± 5.5
γ-Carboxyglutamic acid	12.9 ± 1.7	13.8 ± 1.4
Glutamine	547.0 ± 7.1	558.0 ± 7.7
Proline	36.1 ± 13.1	32.7 ± 1.7
Glycine	163.0 ± 24.7	178.0 ± 21.3
Alanine	283.0 ± 27.9	275.0 ± 43.3
α-Aminobutyric acid	9.1 ± 1.0	5.8 ± 0.7
Valine	188.0 ± 7.9	174.0 ± 9.0
Cysteine	9.2 ± 2.2	5.2 ± 0.1
Methionine	46.5 ± 2.0	40.9 ± 4.4
Isoleucine	92.6 ± 6.4	81.9 ± 8.7
Leucine	257.0 ± 17.9	231.0 ± 25.1
Tyrosine	43.7 ± 5.7	55.7 ± 8.5
Phenylalanine	65.5 ± 1.4	64.5 ± 2.5
Lysine	192.0 ± 15.2	179.0 ± 7.1
Histidine	55.4 ± 2.8	50.9 ± 3.6
3-Methylhistidine	46.1 ± 3.0	43.9 ± 6.8
Arginine	66.9 ± 10.3	61.8 ± 1.7
Taurine	314.0 ± 37.3	418.0 ± 41.2
Citrulline	44.6 ± 7.1	41.8 ± 3.0
Ornithine	30.2 ± 3.7	35.4 ± 4.6
Ammonia	90.8 ± 18.9	127.0 ± 13.7
Uric acid	9954 ± 1389	7737 ± 220

Sera of the wild type and *Decr*$^{-/-}$ mice were collected after 24 h fast. Amino acids were analyzed by the clinical laboratory of the University Hospital of Oulu, Finland. The concentrations are expressed as means ± SEM of 4 mice of each genotype per group.
