# Supplementary material for: Mitochondrial 2,4-dienoyl-CoA Reductase Deficiency in Mice Results in Severe Hypoglycemia with Stress Intolerance and Unimpaired Ketogenesis
Source: PLoS Genet. 2009 Jul 3;5(7):e1000543. doi: 10.1371/journal.pgen.1000543 (PMC2697383; doi:10.1371/journal.pgen.1000543)
Supplement: Table S2 — Urine dicarboxylic acid analysis. Urine of the wild type and Decr−/− mice (5 mice/group) was collected for 24 h (fed sample) and collection was continued for 24 h after food removal (fasted sample). Pooled samples were normalized to urine creatinine and analyzed with mass spectrometry using tetradecanedioic acid (C14:0) as a reference. (0.06 MB PDF) [file pgen.1000543.s003.pdf]

**TABLE S2. Urine dicarboxylic acid analysis**

| | WT | | KO | |
|---|---|---|---|---|
| | Quantity (µmol/l) | | | |
| | fed | fasted | fed | fasted |
| C7:2 | 4.4 | n.d. | n.d. | 20.2 |
| C7:1 | 5.5 | 34.2 | 20.5 | 29.3 |
| C7:0 | 12.5 | 15.2 | 13.6 | 22.6 |
| C8:2 | 16.6 | 39.4 | 50.8 | 154.2 |
| C8:1 | 10.1 | 48.6 | 29.7 | 107.6 |
| C8:0 | 9.8 | 8.4 | 21.1 | 19.1 |
| C9:2 | n.d. | n.d. | n.d. | n.d. |
| C9:1 | 4.5 | 12.8 | 6.0 | 0.0 |
| C9:0 | 20.3 | 28.1 | 19.0 | 8.5 |
| C10:3 | 3.1 | 4.5 | 16.5 | 54.6 |
| C10:2 | n.d. | 7.9 | 51.4 | 72.1 |
| C10:1 | 6.8 | 13.0 | 30.4 | 33.4 |
| C10:0 | 27.6 | 35.4 | 58.6 | 43.8 |
| C11:3 | 2.5 | n.d. | n.d. | 2.8 |
| C11:2 | n.d. | n.d. | n.d. | n.d. |
| C11:1 | 3.6 | 4.1 | n.d. | n.d. |
| C11:0 | 6.0 | 7.0 | 6.7 | 21.7 |
| C12:3 | n.d. | n.d. | n.d. | n.d. |
| C12:2 | n.d. | n.d. | n.d. | n.d. |
| C12:1 | 13.2 | 11.3 | 7.8 | 13.5 |
| C12:0 | 4.4 | n.d. | n.d. | n.d. |
| C13:3 | 4.7 | n.d. | n.d. | n.d. |
| C13:2 | n.d. | n.d. | n.d. | 7.7 |
| C13:1 | n.d. | n.d. | n.d. | n.d. |
| C13:0 | n.d. | 14.3 | n.d. | 10.3 |
| C14:3 | n.d. | n.d. | 16.0 | 16.2 |
| C14:2 | n.d. | n.d. | n.d. | n.d. |
| C14:1 | n.d. | n.d. | n.d. | n.d. |
| C14:0 | 50.0 | 50.0 | 50.0 | 50.0 |
| C15:0 | n.d. | n.d. | n.d. | n.d. |
| C16:0 | n.d. | n.d. | n.d. | n.d. |

Urine of the wild type and *Decr*$^{-/-}$ mice (5 mice/group) was collected for 24 h (fed sample) and collection was continued for 24h after food removal (fasted sample). Pooled samples were normalized to urine creatinine and analyzed with mass spectrometry using tetradecanedioic acid (C14:0) as a reference.
